# Supplementary material for: Cannabis consumption is associated with altered steroid metabolism in young men
Source: Commun Med (Lond). 2026 Apr 16;6:224. doi: 10.1038/s43856-026-01469-x (PMC13087242; doi:10.1038/s43856-026-01469-x)
Supplement: Supplementary file 2 — Supplementary Information [file 43856_2026_1469_MOESM2_ESM.pdf]

Cannabis consumption is associated with altered steroid metabolism in young men

Mathieu Galmiche, Isabel Meister, Fanny Zufferey, Michel F. Rossier, Rita Rahban, Alfred Senn, Serge Nef, Julien Boccard, Serge Rudaz

Supporting Information

Section S1: Extended steroid profiling in the cohort of 94 male human serum samples 4

Section S2: OPLS-DA model (M1) discriminating the circulating steroid profile from cannabis users and non-users 5

Section S3: Univariate analyses of C11-oxy androgen concentrations depending on the cannabis consumption status 7

Section S4: PLS model (M2) relating the circulating steroid profile with serum THC concentrations in cannabis users.. 8

Section S5: PLS model (M3) relating the circulating steroid profile with serum THC-COOH concentrations in cannabis users 11

Section S6: Univariate analyses comparing androgen concentrations between occasional and chronic cannabis consumers.. 13

Section S7: OPLS-DA model (M4) discriminating the circulating steroid profile from chronic and occasional cannabis users 14

Section S8: Investigation of possible confounding factors in the study of the extended steroid profile in relation with cannabis usage 16

Section S9: Correlation of the steroid profile with other sex hormones... 19

Section S10: Method for THC and THC-COOH determination in serum...................................... 20

Figure S 1 : Histogram representing the variability of normalized peak areas measured for each detected compound in pooled QC samples........ 4
Figure S 2 : Partition of the detected steroid compounds building up the extended steroid profile dataset within the eight defined steroid subclasses........ 4
Figure S 3 : Scores plot showing the separation of samples from the “THC-positive” group and the “control” group according to the OPLS-DA model M1........ 5
Figure S 4 : Loadings plot displaying the variables responsible for group separation in model M1.. 5
Figure S 5 : Variables Importance in Projection (VIP) characterizing the importance of each variable in the predictive component of model M1........ 6
Figure S 6 : Model M1 permutation plot displaying observed R^2Y and Q^2 versus corresponding values obtained under the null hypothesis in a permutation test with 100 permutations. 6
Figure S 7 : Beeswarm charts with univariate statistical analyses (t-tests), showing the differences in circulating C11-oxy androgen concentrations between controls and THC-positive participants ... 7
Figure S 8 : Observed vs. predicted values of serum THC concentrations according to the PLS model M2 8
Figure S 9 : Loadings plot showing the distribution of variables from the steroid profile (X-variables) and serum THC concentration (Y-variable) in the PLS model M2........ 8
Figure S 10 : Regression coefficients representing the contribution of each X-variable from the extended steroid profile to predict the serum THC concentration (Y-variable) in the PLS model M2. 9
Figure S 11 : Variables Importance in Projection (VIP) characterizing the importance of each X-variable in the prediction of the serum THC concentration (Y) in M2. 9
Figure S 12 : Annotated loadings plot from M2 model showing that gonadal androgens (A4, T and DHT) are positively correlated with serum THC concentrations, whereas adrenal androgens (11B-OHA4, 11B-OHT, 11-KA4, 11-KT) are rather not correlated or negatively correlated with serum THC. 10
Figure S 13 : Observed vs. predicted values of serum THC-COOH levels according to the PLS model M3 11
Figure S 14 : Loadings plot showing the distribution of variables from the steroid profile (X-variables) and serum THC concentration (Y-variable) in the PLS model M3........ 11
Figure S 15 : Regression coefficients representing the contribution of each X-variable from the extended steroid profile to predict the serum THC-COOH concentration (Y-variable) in the PLS model M3. 12
Figure S 16 : Variables Importance in Projection (VIP) characterizing the importance of each X-variable in the prediction of the serum THC-COOH concentration (Y) in M3. 12
Figure S 17 : Beeswarm charts with univariate statistical analyses (t-tests) showing the differences in steroid concentrations between occasional (light green) and chronic (dark green) cannabis consumers. 13

Figure S 18 : Scores plot showing the separation of samples from the occasional and the chronic consumer groups according to the OPLS-DA model M4. 14
Figure S 19 : Loadings plot displaying the variables responsible for group separation in model M4 14
Figure S 20 : Variables Importance in Projection (VIP) characterizing the importance of each variable in the predictive component of model M5. 15
Figure S 21 : Model M5 permutation plot displaying observed R^2Y and Q^2 versus corresponding values obtained under the null hypothesis in a permutation test with 100 permutations. 15
Figure S 22 : Beeswarm chart with the corresponding univariate statistical analysis (two-tailed t-test) demonstrating the absence of significant difference in sampling time between the subjects of each group (THC-positive vs. control).. 16
Figure S 23 : Beeswarm chart with the corresponding univariate statistical analysis (two-tailed t-test) demonstrating the absence of significant difference in Body Mass Index between the subjects of each group (THC-positive vs. control) 18
Figure S 24 : Estimation plots from univariate statistical analyses (t-tests), showing the differences in circulating levels of A) luteinizing hormone (LH) and B) follicle stimulating hormone (FSH) between controls and THC-positive participants (left pane), or occasional and chronic cannabis consumers (right pane). 19

Section S1: Extended steroid profiling in the cohort of 94 male human serum samples

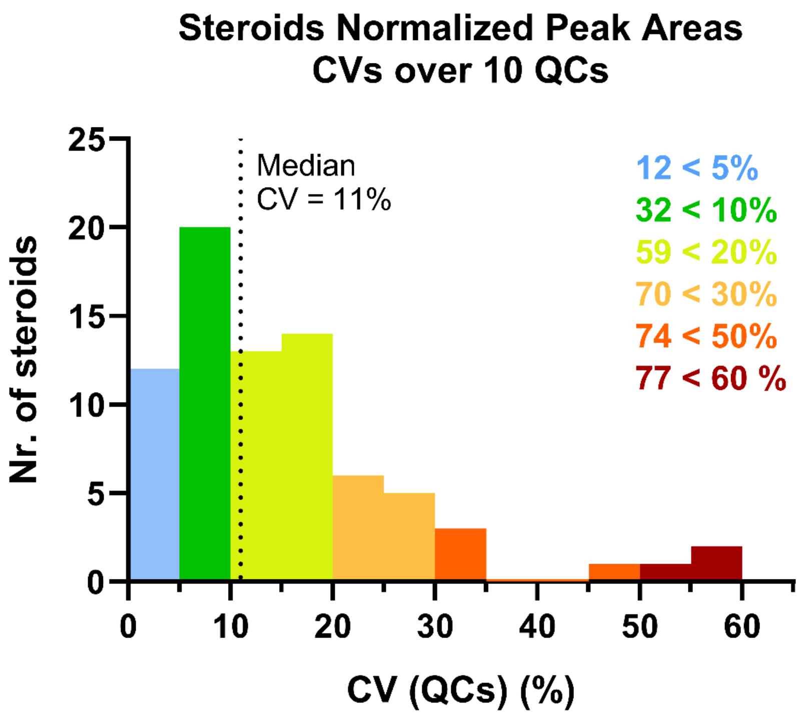

Figure S 1 : Histogram representing the variability of normalized peak areas measured for each detected compound in pooled QC samples

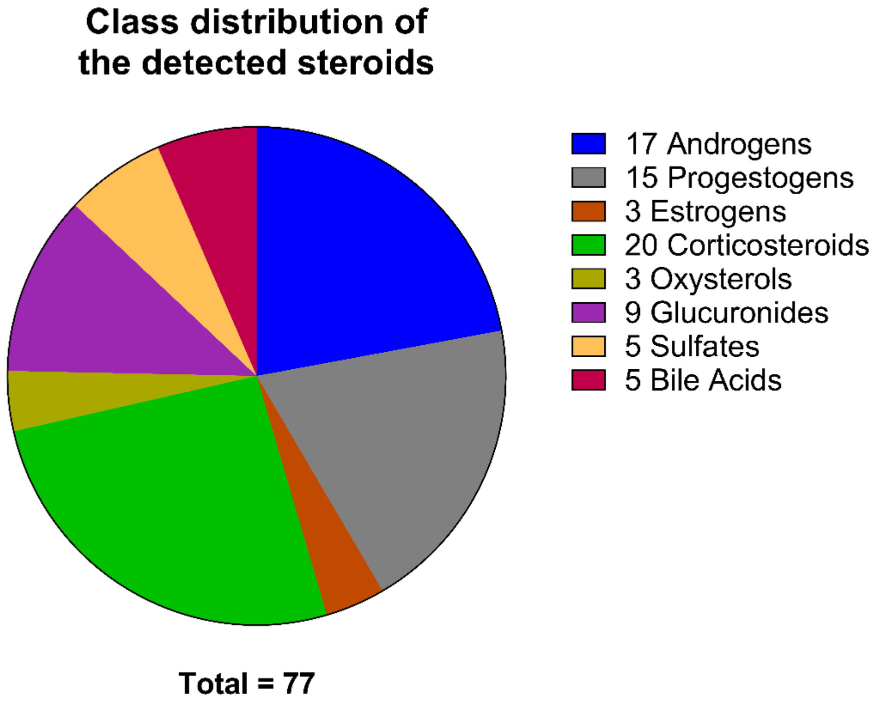

Figure S 2 : Partition of the detected steroid compounds building up the extended steroid profile dataset within the eight defined steroid subclasses

Section S2: OPLS-DA model (M1) discriminating the circulating steroid profile from cannabis users and non-users

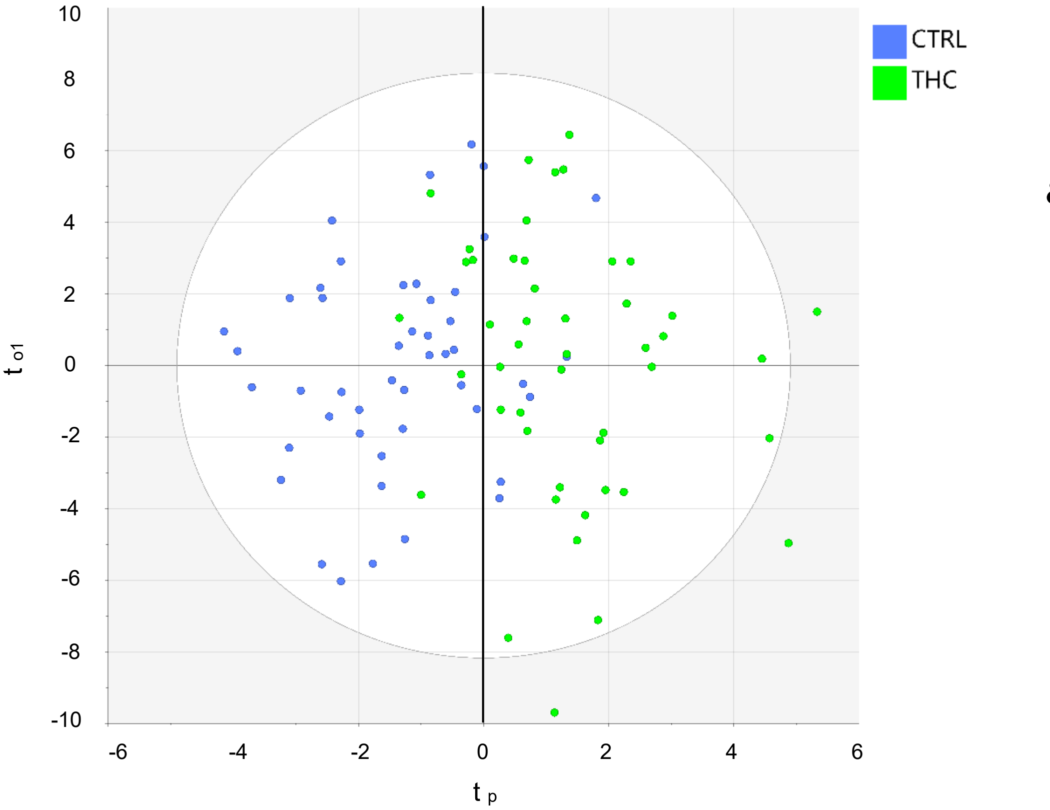

M1

1 predictive

& 1 orthogonal

component

$R^2Xp = 0.055$

$R^2Y = 0.49$

$Q^2 = 0.19$

Figure S 3 : Scores plot showing the separation of samples from the "THC-positive" group and the "control" group according to the OPLS-DA model M1.

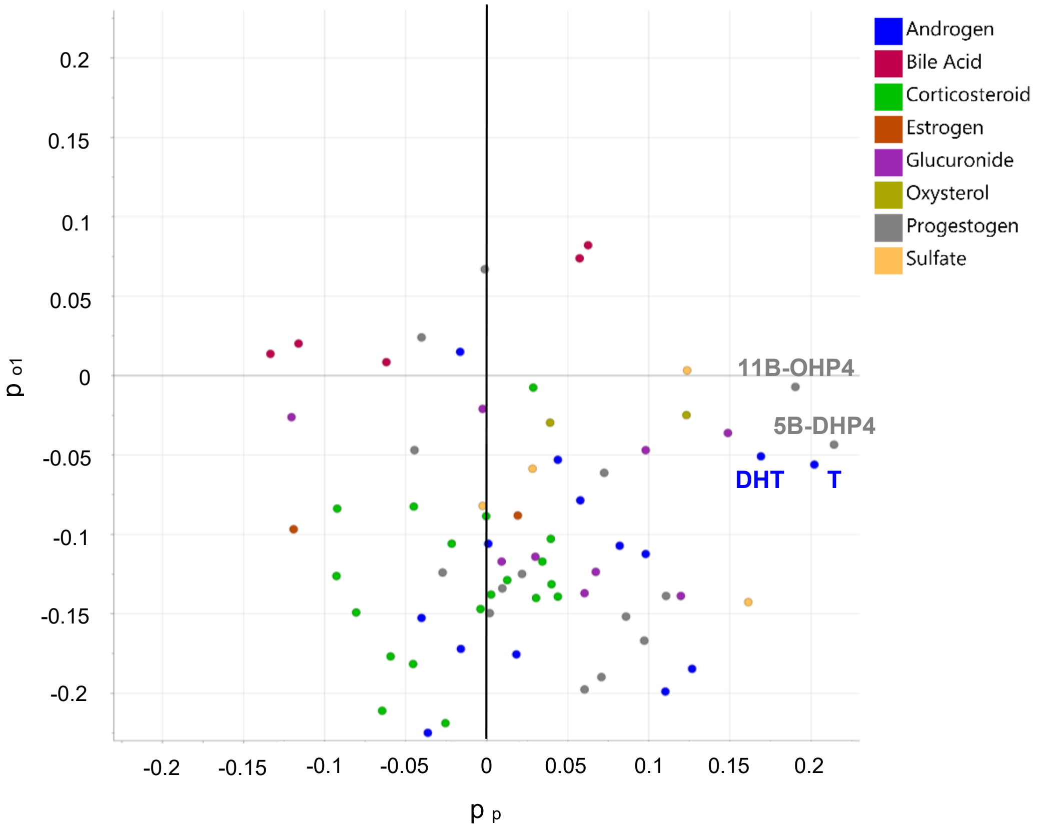

Figure S 4 : Loadings plot displaying the variables responsible for group separation in model M1

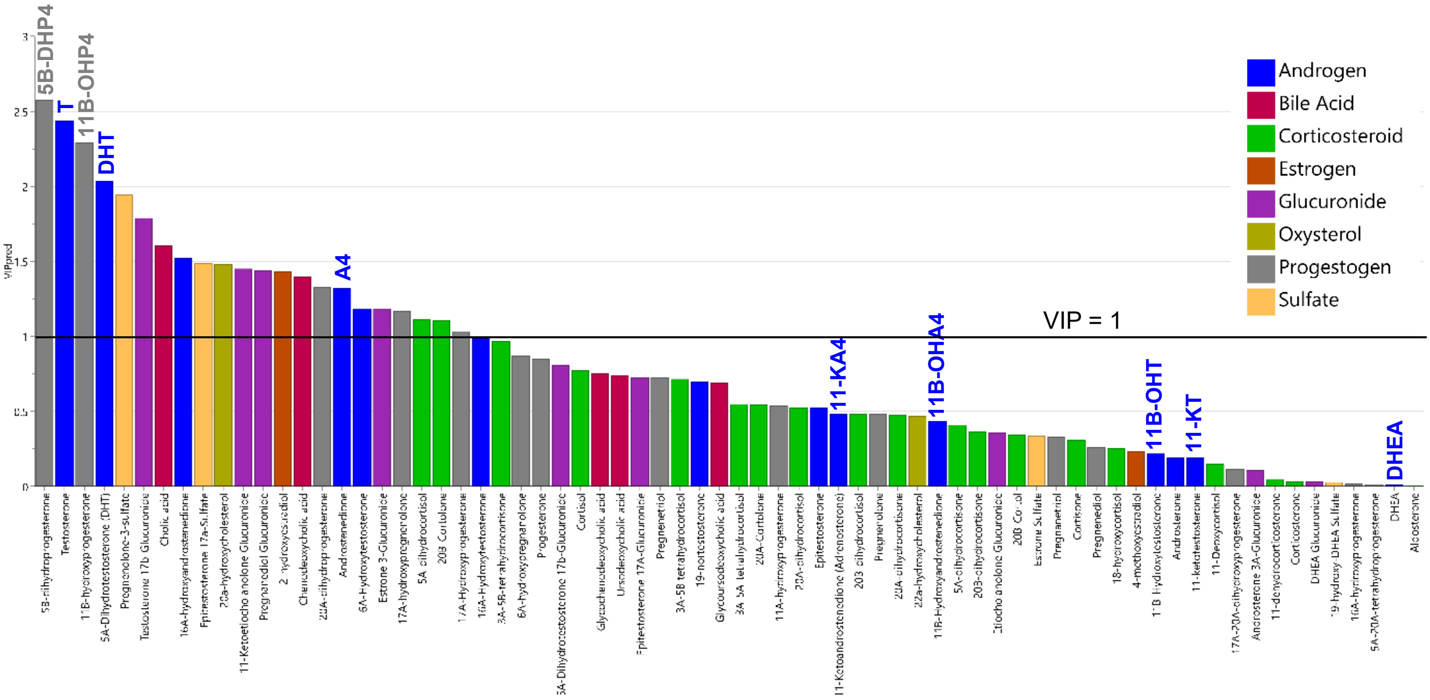

Figure S 5 : Variables Importance in Projection (VIP) characterizing the importance of each variable in the predictive component of model M1.

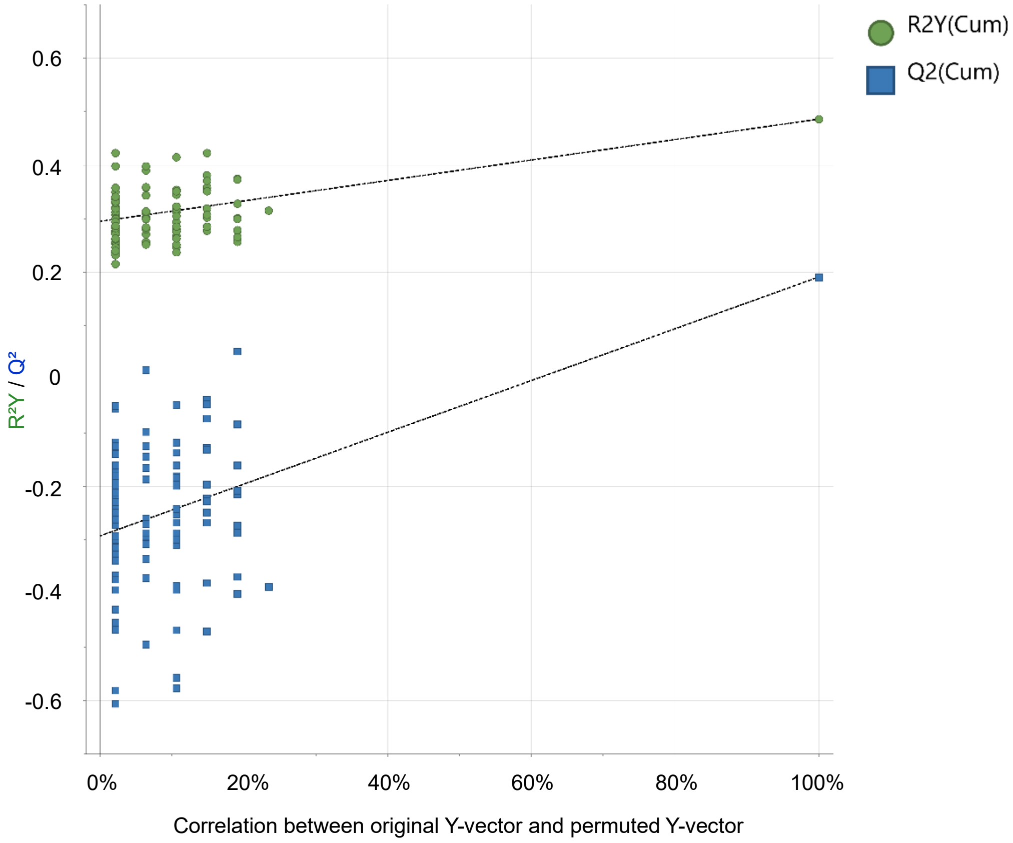

Figure S 6 : Model M1 permutation plot displaying observed R²Y and Q² versus corresponding values obtained under the null hypothesis in a permutation test with 100 permutations.

Section S3: Univariate analyses of C11-oxy androgen concentrations depending on the cannabis consumption status

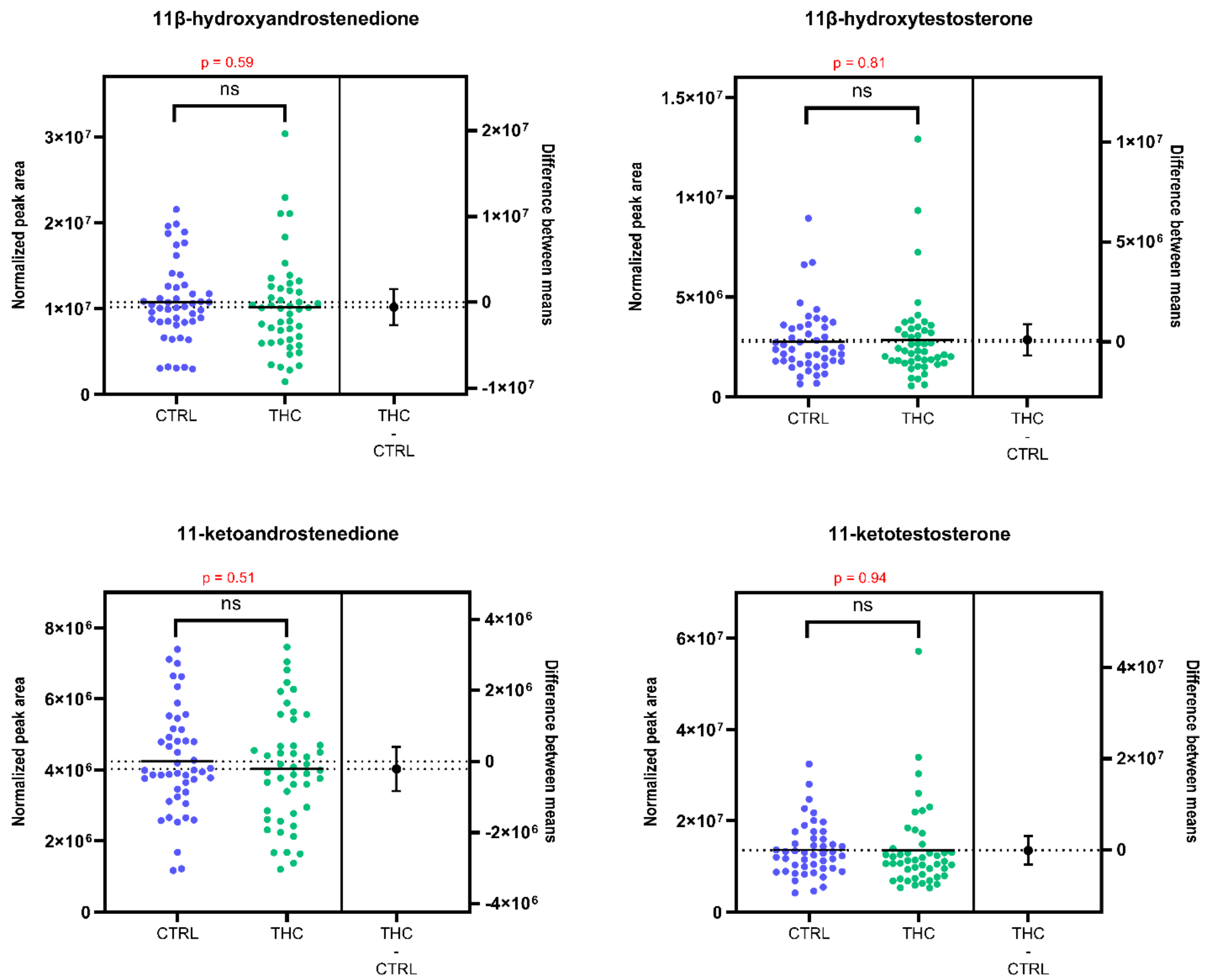

Figure S 7 : Beeswarm charts with univariate statistical analyses (t-tests), showing the differences in circulating C11-oxy androgen concentrations between controls and THC-positive participants

Section S4: PLS model (M2) relating the circulating steroid profile with serum THC concentrations in cannabis users

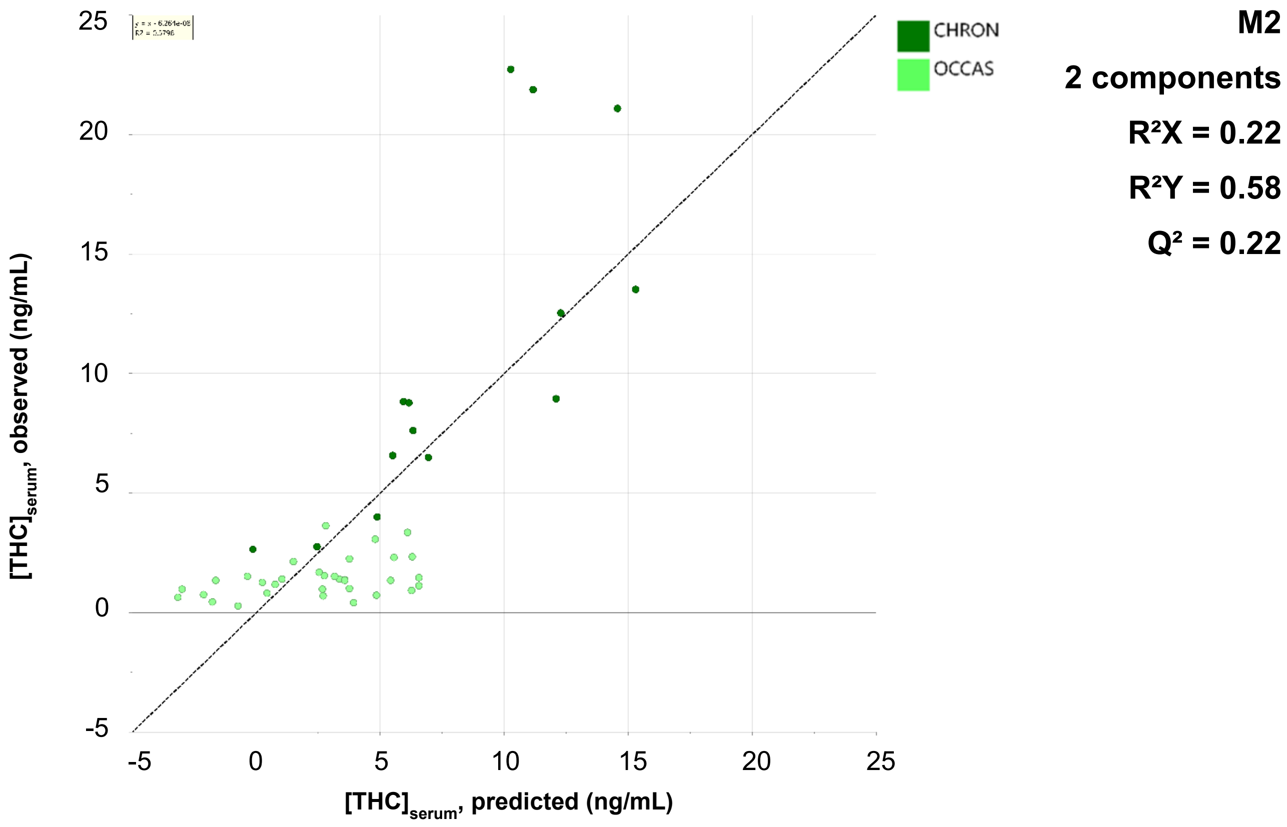

Figure S 8 : Observed vs. predicted values of serum THC concentrations according to the PLS model M2

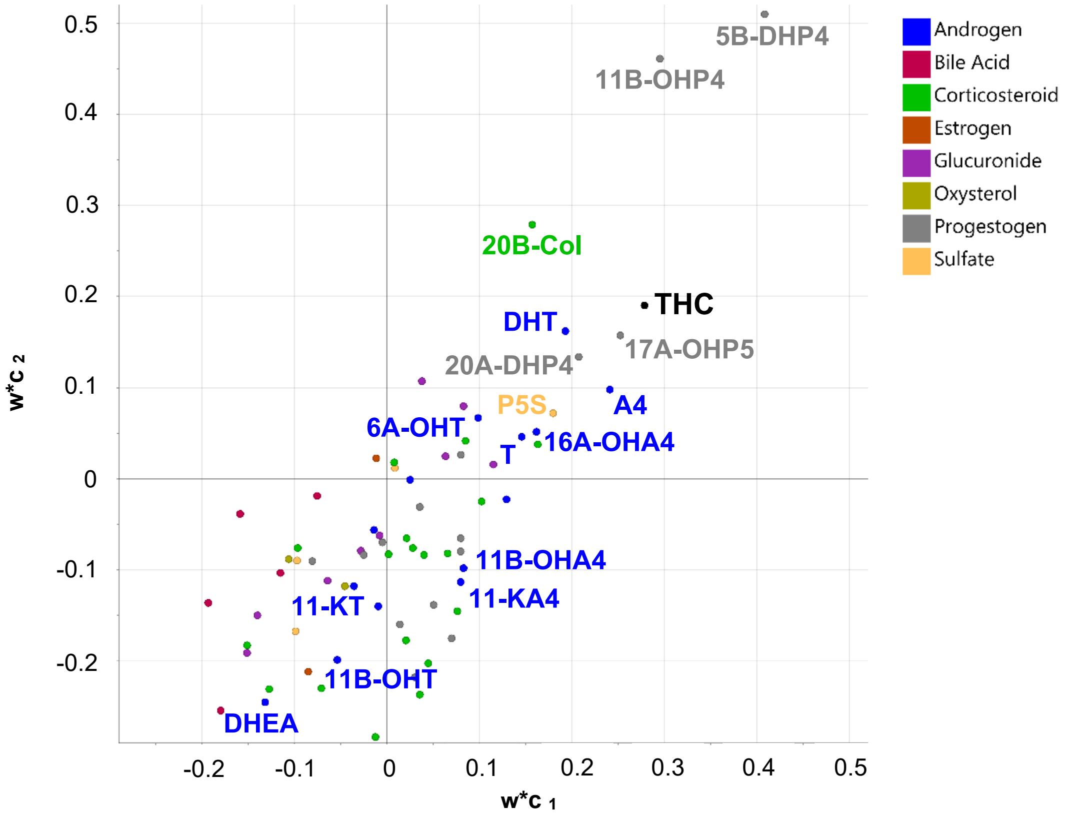

Figure S 9 : Loadings plot showing the distribution of variables from the steroid profile (X-variables) and serum THC concentration (Y-variable) in the PLS model M2.

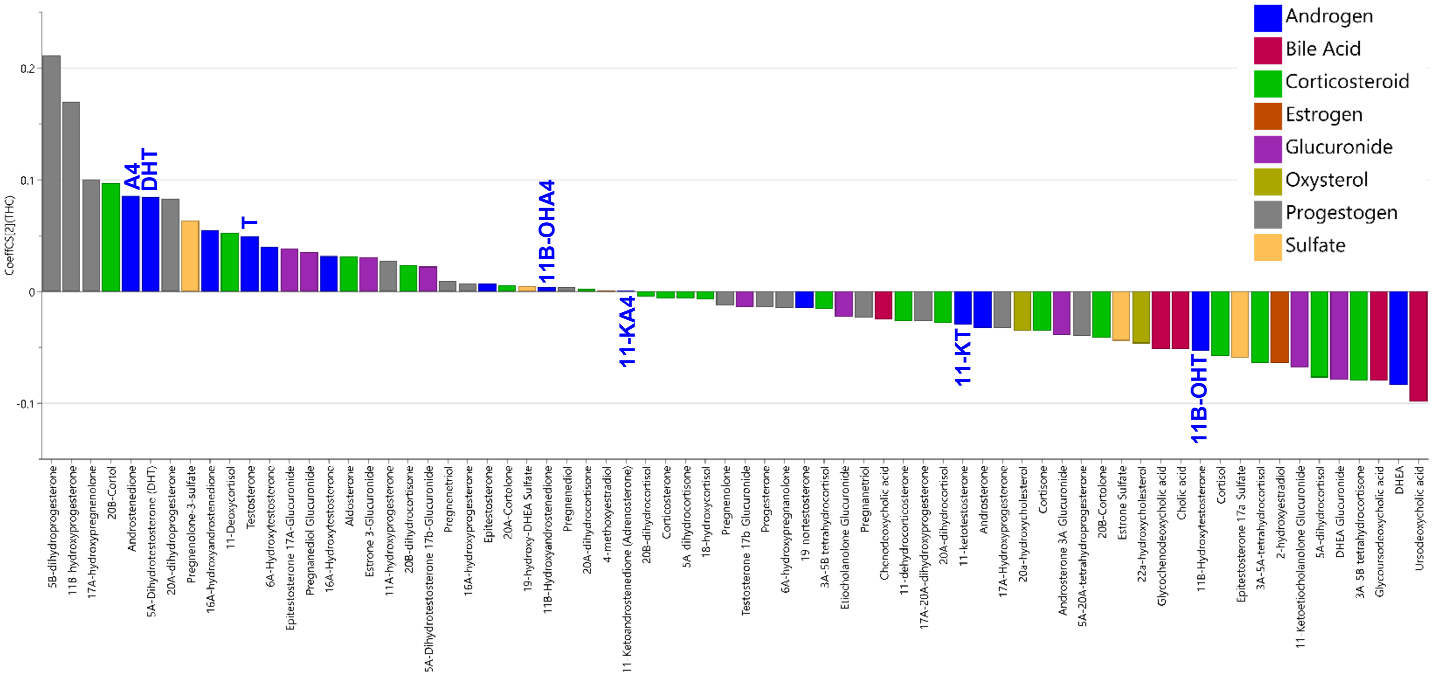

Figure S 10 : Regression coefficients representing the contribution of each X-variable from the extended steroid profile to predict the serum THC concentration (Y-variable) in the PLS model M2.

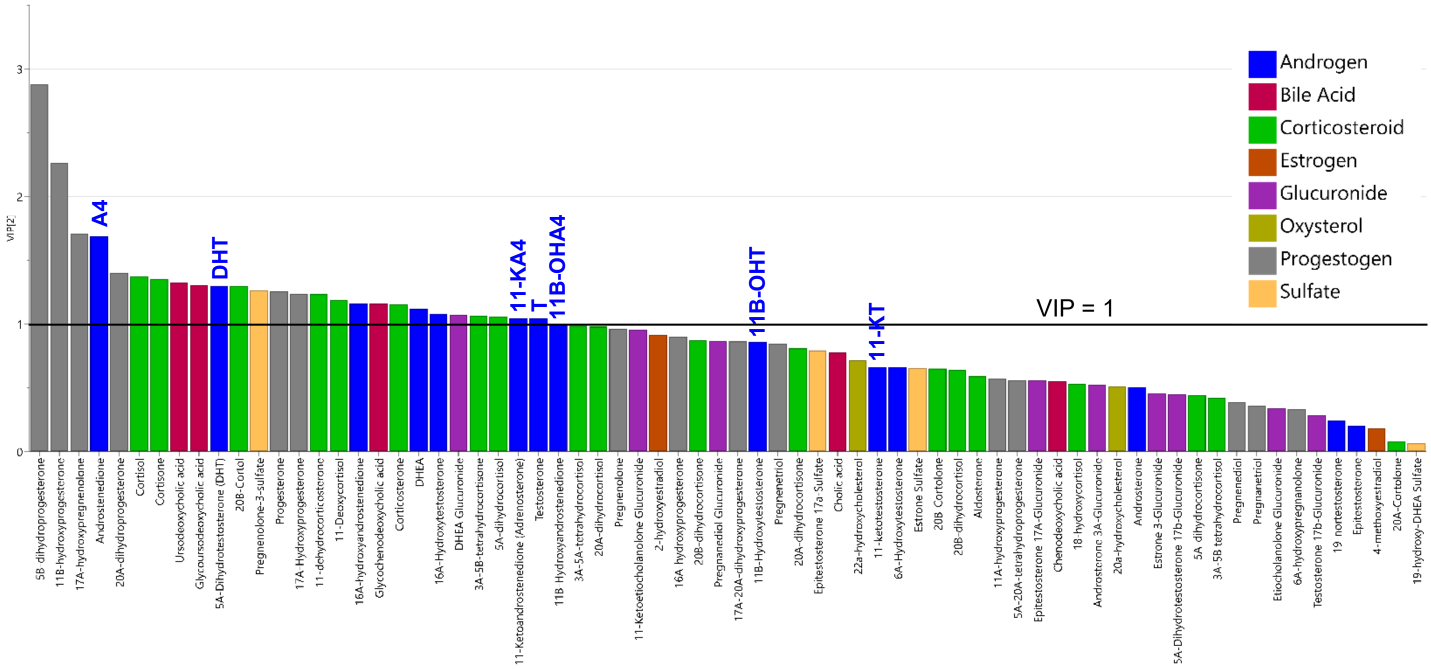

Figure S 11 : Variables Importance in Projection (VIP) characterizing the importance of each X-variable in the prediction of the serum THC concentration (Y) in M2.

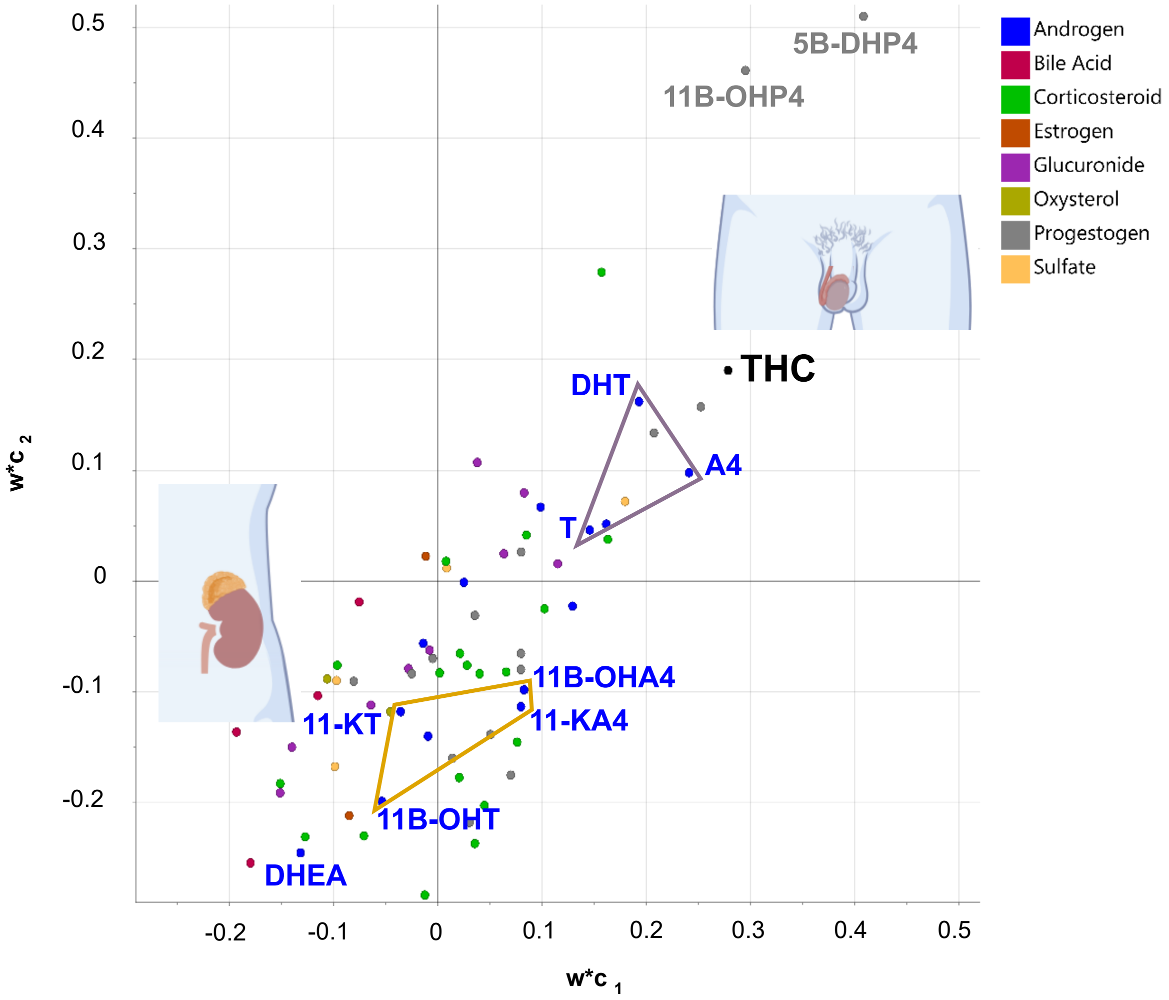

Figure S 12 : Annotated loadings plot from M2 model showing that gonadal androgens (A4, T and DHT) are positively correlated with serum THC concentrations, whereas adrenal androgens (11B-OHA4, 11B-OHT, 11-KA4, 11-KT) are rather not correlated or negatively correlated with serum THC.

Section S5: PLS model (M3) relating the circulating steroid profile with serum THC-COOH concentrations in cannabis users

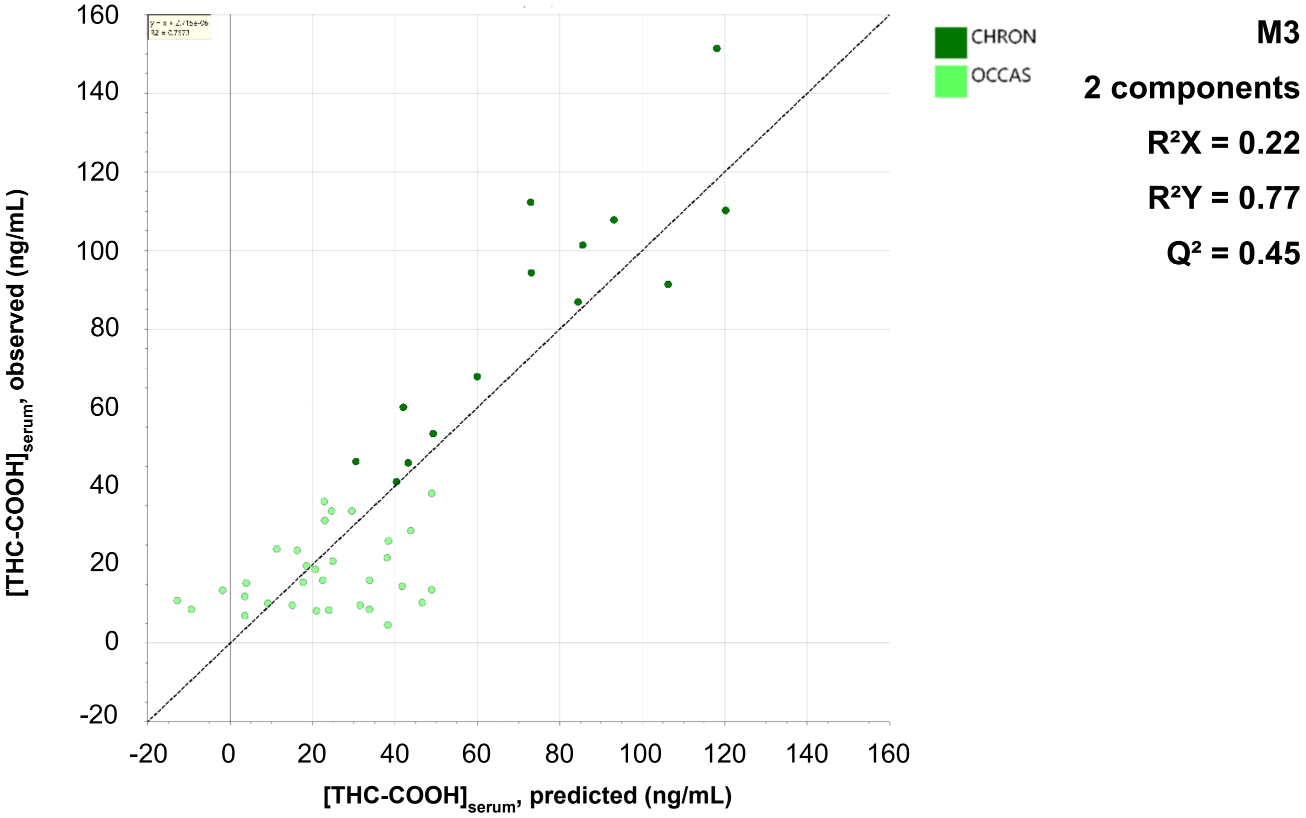

Figure S 13 : Observed vs. predicted values of serum THC-COOH levels according to the PLS model M3

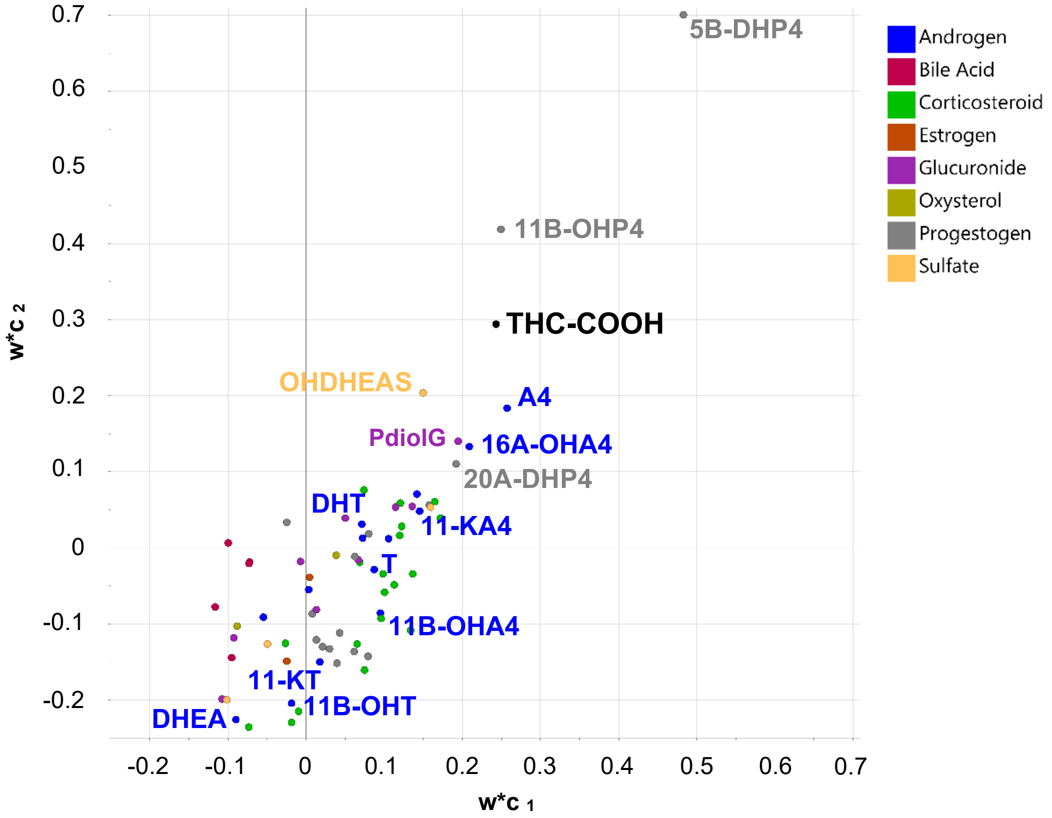

Figure S 14 : Loadings plot showing the distribution of variables from the steroid profile (X-variables) and serum THC concentration (Y-variable) in the PLS model M3.

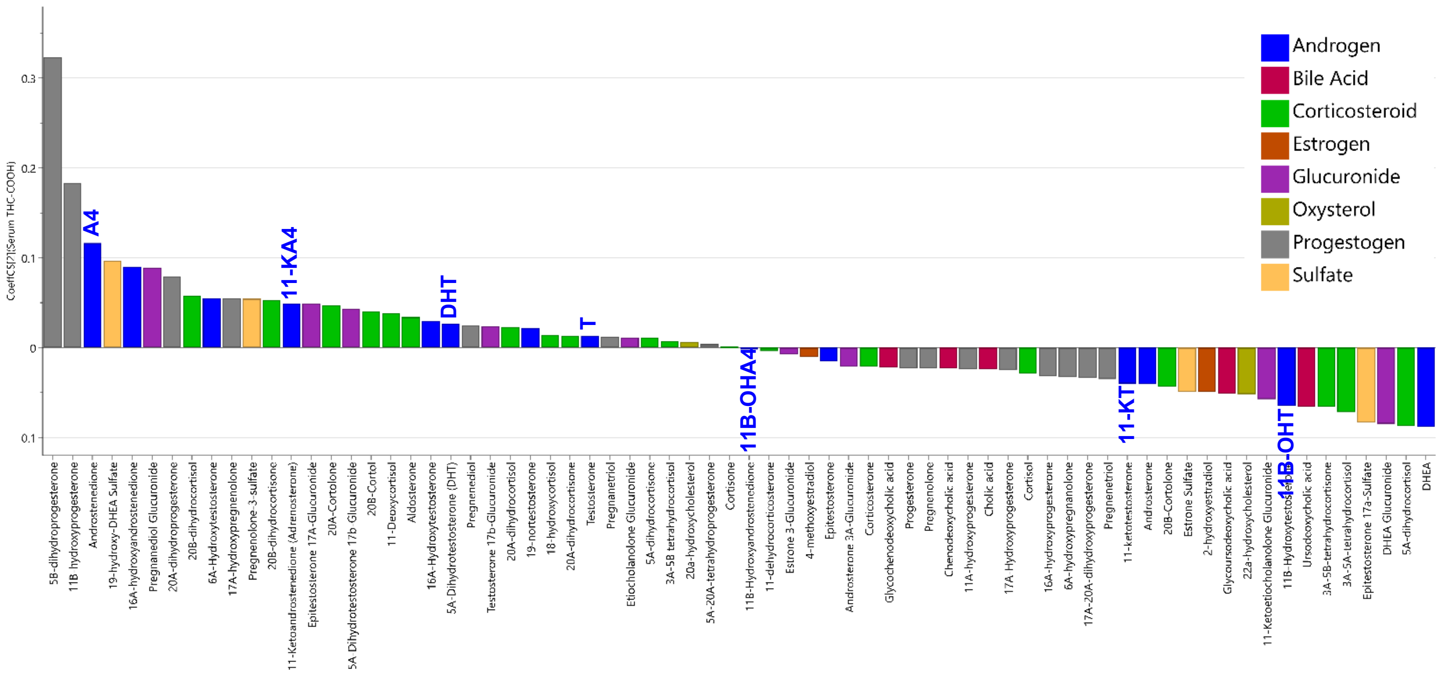

Figure S 15 : Regression coefficients representing the contribution of each X-variable from the extended steroid profile to predict the serum THC-COOH concentration (Y-variable) in the PLS model M3.

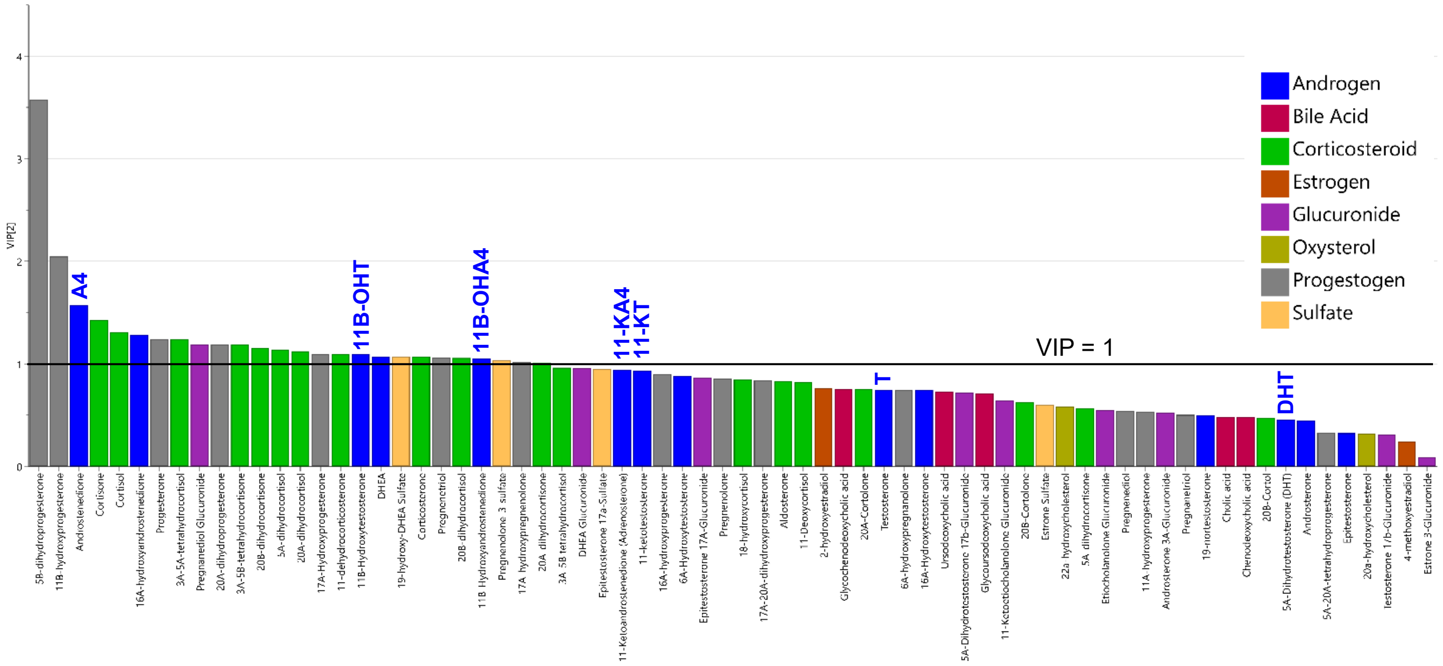

Figure S 16 : Variables Importance in Projection (VIP) characterizing the importance of each X-variable in the prediction of the serum THC-COOH concentration (Y) in M3.

Section S6: Univariate analyses comparing androgen concentrations between occasional and chronic cannabis consumers

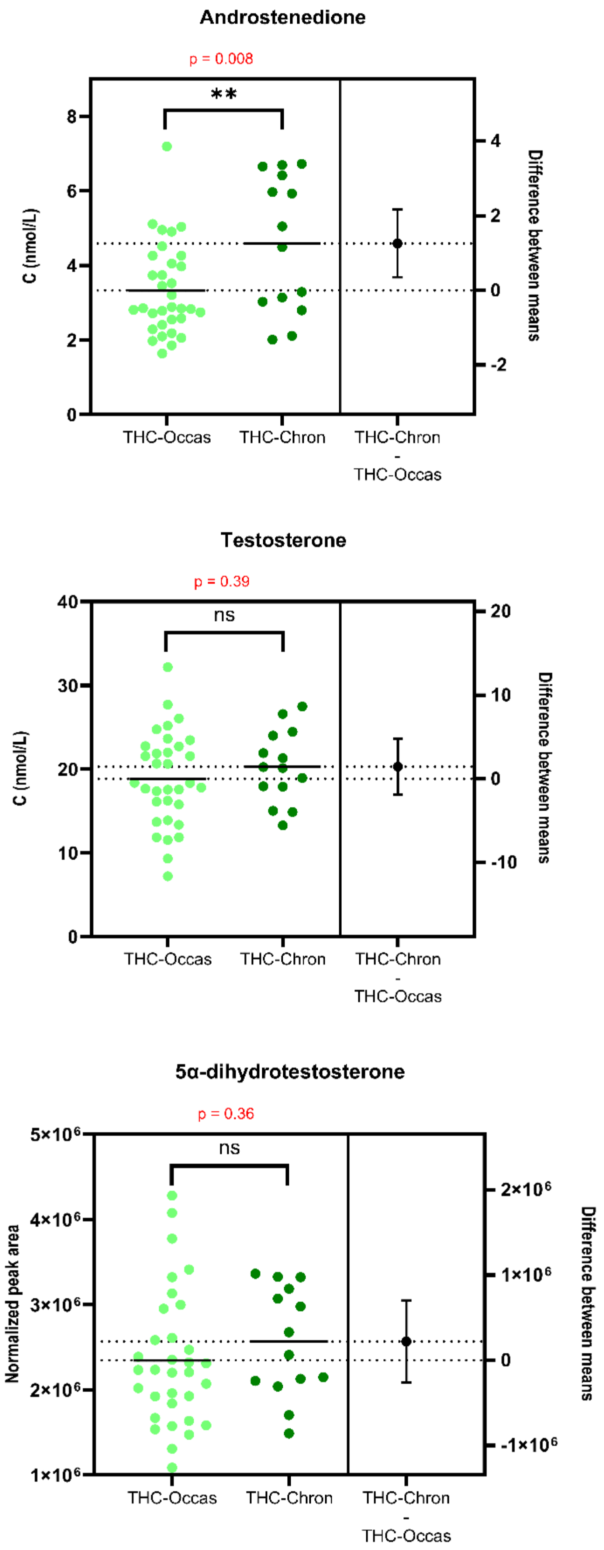

Figure S 17: Beeswarm charts with univariate statistical analyses (t-tests) showing the differences in steroid concentrations between occasional (light green) and chronic (dark green) cannabis consumers.

Section S7: OPLS-DA model (M4) discriminating the circulating steroid profile from chronic and occasional cannabis users

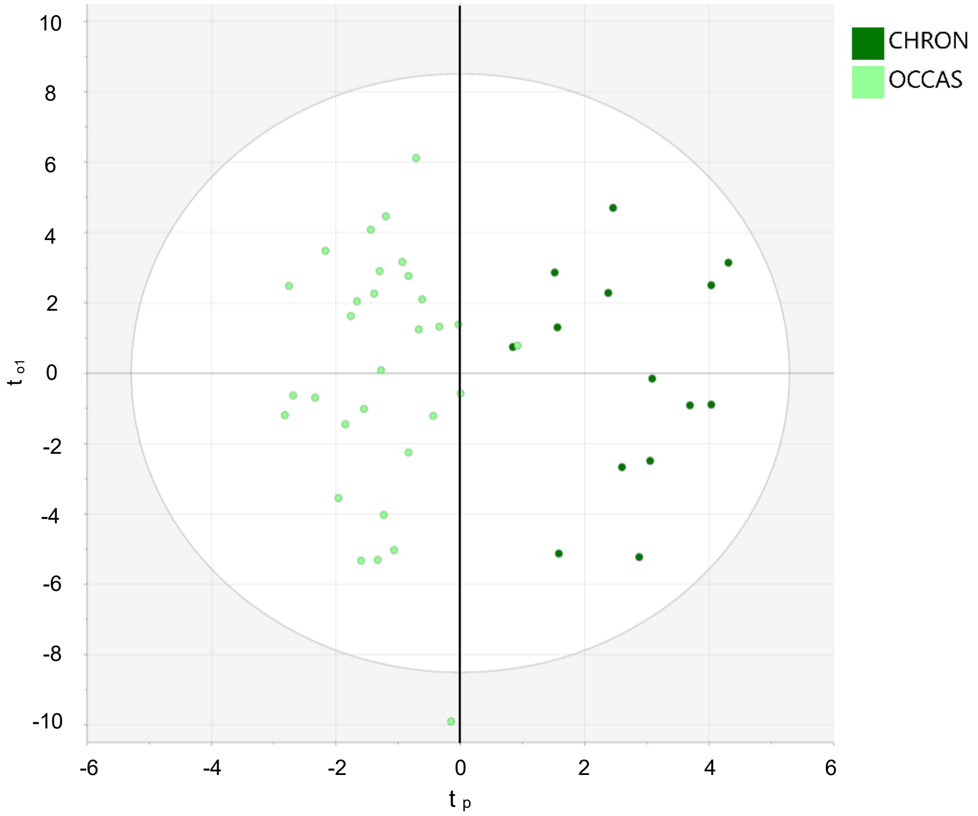

M4
1 predictive &
2 orthogonal
components
$R^2Xp = 0.061$
$R^2Y = 0.80$
$Q^2 = 0.36$

Figure S 18 : Scores plot showing the separation of samples from the occasional and the chronic consumer groups according to the OPLS-DA model M4.

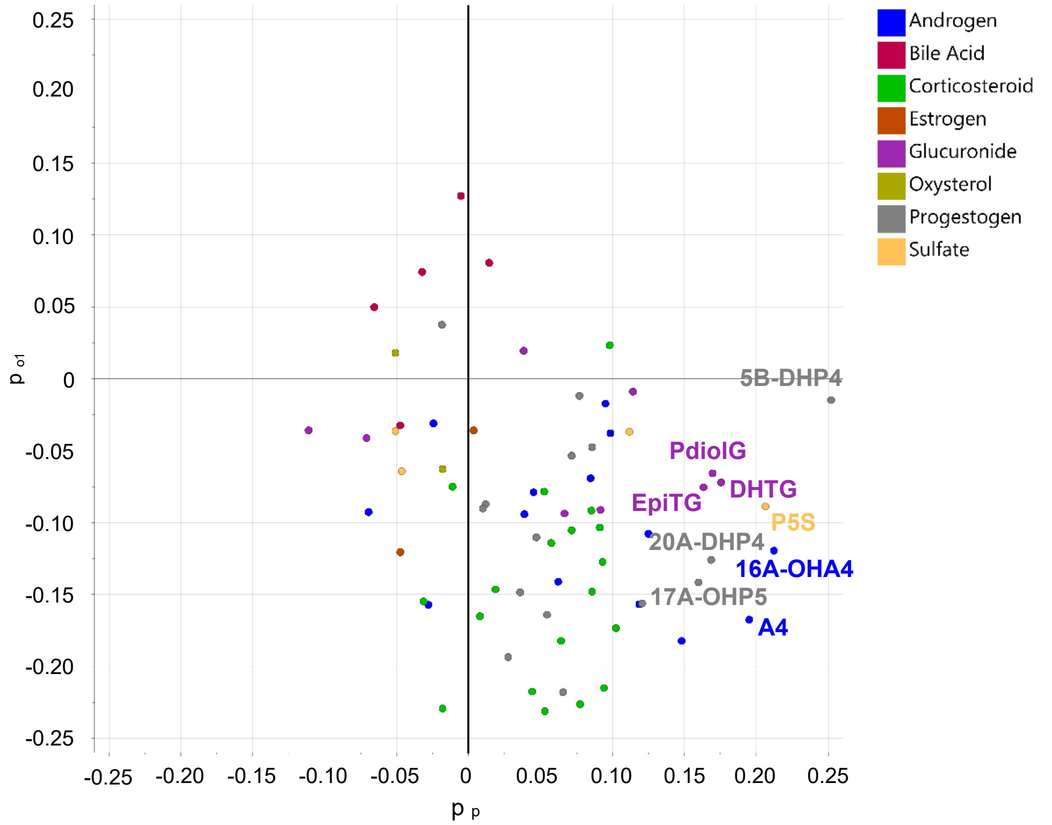

Figure S 19 : Loadings plot displaying the variables responsible for group separation in model M4

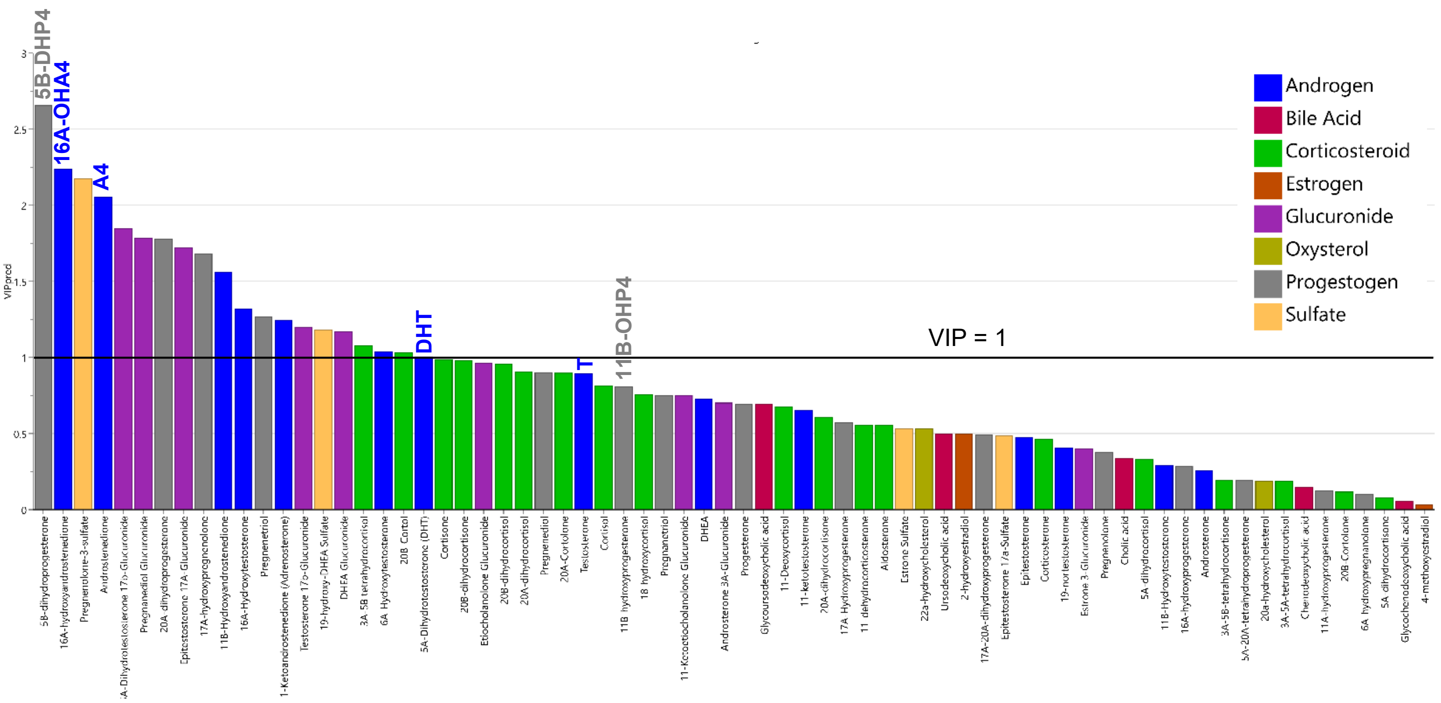

Figure S 20 : Variables Importance in Projection (VIP) characterizing the importance of each variable in the predictive component of model M5.

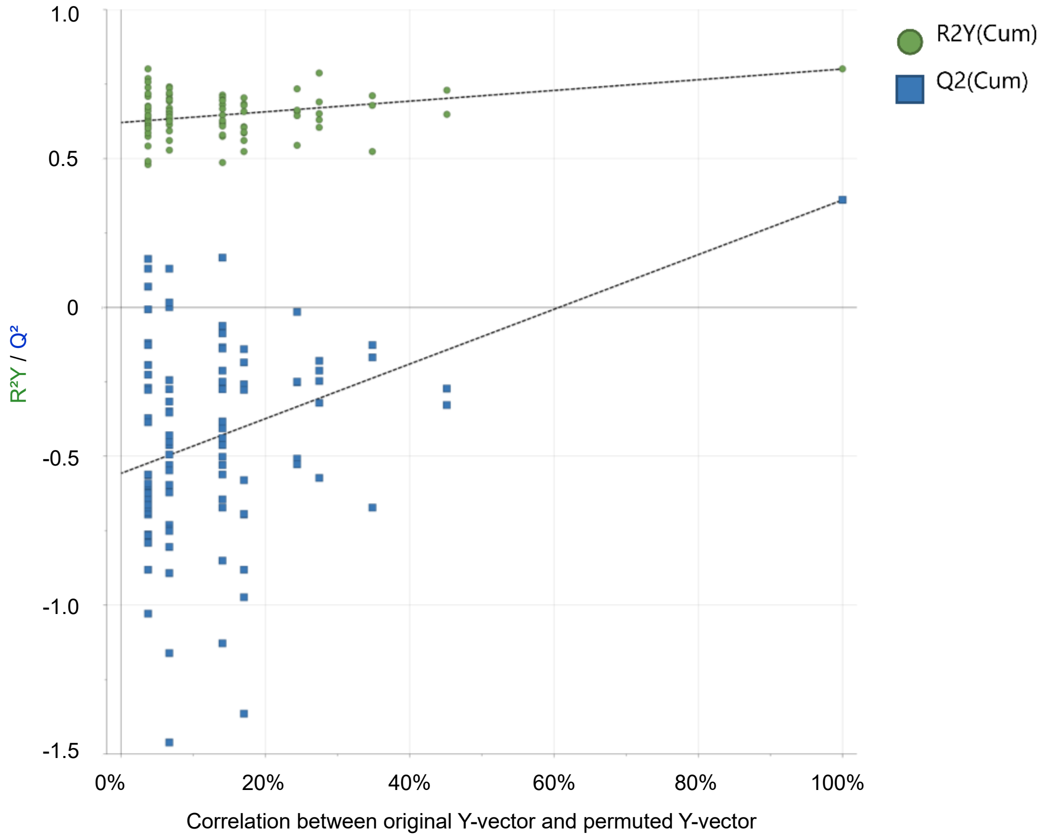

Figure S 21 : Model M5 permutation plot displaying observed R²Y and Q² versus corresponding values obtained under the null hypothesis in a permutation test with 100 permutations.

Section S8: Investigation of possible confounding factors in the study of the extended steroid profile in relation with cannabis usage

In this study, three potential confounding factors were identified *a priori*, and could be investigated with the available data: time of day at sampling, tobacco consumption and Body Mass Index (BMI).

a. Time of day at sampling

For some steroid metabolites, circulating concentrations in blood display strong diurnal variations (Collomp *et al.*, 2016). In this study, it was therefore crucial to ensure consistence in the time of day at blood sampling for each participant. When possible, blood samples were taken in the late afternoon. Although there were some discrepancies in sampling time of different participants, this variable was homogeneous between the 'THC-positive' group and the control group (p = 0.41, see Figure S22). Cortisol, which is the most affected steroid with respect to circadian rhythm, was quantified at very similar levels in the two groups (p = 0.72, see Table 1 of the Manuscript). Furthermore, when outlier concentrations were measured for steroids such as androstenedione and testosterone, they did not correspond to participants with outlier sampling times. Instead, they corresponded to samples taken close to the mean time of sampling of the study. Altogether, these data suggest that blood sampling time was not a significant confounder in the present study.

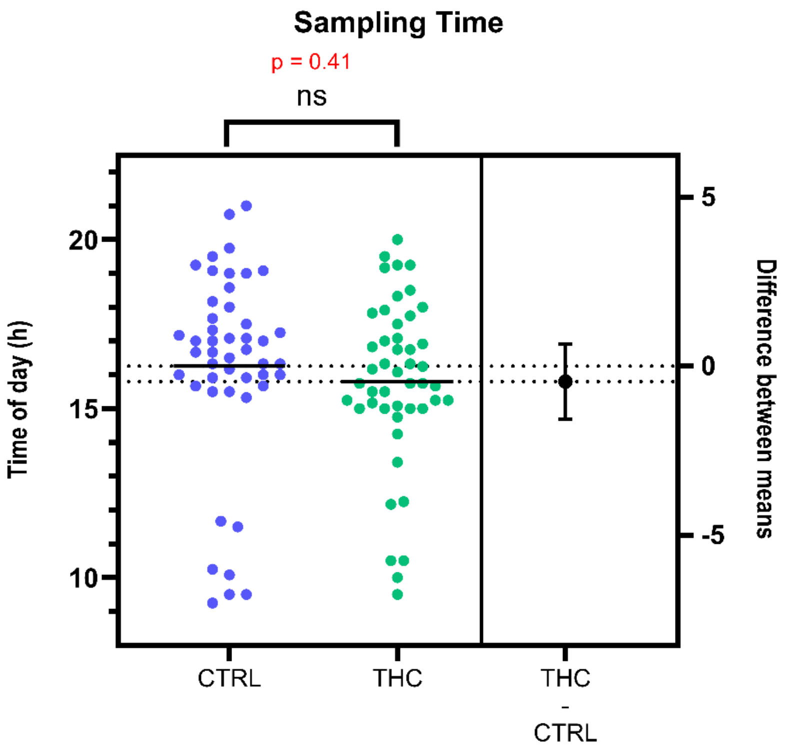

Figure S 22 : Beeswarm chart with the corresponding univariate statistical analysis (two-tailed t-test) demonstrating the absence of significant difference in sampling time between the subjects of each group (THC-positive vs. control)

b. Tobacco consumption

Tobacco smoking is known to be a major confounder in studies aiming at deciphering the effects of cannabis on male reproductive health (Shiels *et al.*, 2009 ; Gundersen *et al.,* 2015 ; Deltourbe *et al.,* 2025). It is indeed frequent that cannabis smokers are also tobacco smokers. Gundersen *et al.* could not separate variations of testosterone levels that would be solely due to marijuana use from the influence of cigarette smoking (Gundersen *et al.,* 2015).

In our cohort, there was also an important overlap between participants who declared cannabis smoking and cigarette smoking. Only 10 out of 47 controls smoked tobacco, and only 5 out of 47 THC-positive participants did not smoke tobacco. In total, there were 52 cigarette smokers among the 94 participants of the study:

	**Cigarette smokers**	**Non-smokers**
**Cannabis users (THC-positive)**	42	5
**Controls (THC-negative)**	10	37
**Total**	52	42

The OPLS-DA model differentiating cigarette smokers (n = 52) from non-smokers (n = 42) displayed worse metrics than the models based on cannabis consumption ($R^2Y = 0.41$ and $Q^2 = -0.01$ with two components). The negative value of Q^2, along with the permutation test that shows several Q^2 values obtained under the null hypothesis higher than the experimental one, suggests that in the present dataset, the prediction of cigarette smoking status based on steroid profile is unreliable. Therefore, although some discriminant variables of this tobacco-based OPLS-DA model are in common with cannabis-related models (e.g., 5B-DHP4), **the effects observed on the extended steroid profile in this cohort and described in this manuscript are primarily related to the cannabis smoking status, not to the cigarette smoking status.**

This was confirmed by the attempt to generate a PLS model with quantitative information on the number of cigarettes consumed daily by cigarette smokers, as they declared it. This attempt was unsuccessful, as the Q^2 value of the model was negative and decreasing at each additional component. Again, the steroid profile measured in this cohort was not related to the amount of tobacco smoking. As a drawback, the tobacco consumption of the participants was only evaluated from their statement and was not confirmed by any biomarker, oppositely to the cannabis consumption which was confirmed by THC and THC-COOH.

c. Body Mass Index

BMI is known to impact circulating steroid concentrations in males (Mezzullo *et al.,* 2020 ; Deltourbe *et al.,* 2025). In the present study, we ensured that the THC-positive and control groups were matched in BMI, as no significant difference in BMI was observed between the two groups (p-value = 0.69, see Figure S23). Furthermore, there was no model able to relate steroid profile as X-variable to BMI as Y-variable using PLS regression. No component at all was found to be significant in this attempt to generate a BMI-based model ($Q^2 < 0$ starting from the first component and decreasing at each additional component). Therefore, it was determined that **BMI was not a significant confounding factor in the present study**.

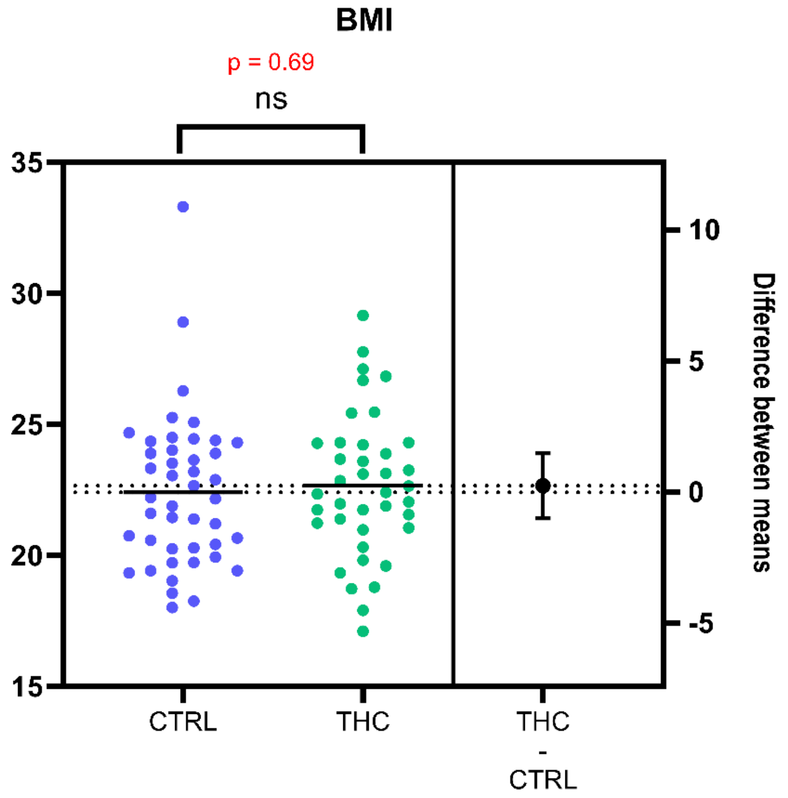

Figure S 23 : Beeswarm chart with the corresponding univariate statistical analysis (two-tailed t-test) demonstrating the absence of significant difference in Body Mass Index between the subjects of each group (THC-positive vs. control)

Section S9: Correlation of the steroid profile with other sex hormones

Data on the extended steroid profile measured in participants from this cohort were combined with data on LH and FSH measured in the same samples by Zufferey *et al.* [20]. However, no robust PLS model could be established to relate these hormones secreted by the anterior pituitary to circulating steroid concentrations. Q^2 values were negative for both multivariate models, irrespective of the number of components involved. The dataset was even reduced to sex hormones only (androgens, progestogens, estrogens and their phase II metabolites) in an attempt to reduce the noise related to other steroid subclasses, without yielding any improvement.

LH was slightly, insignificantly increased in cannabis users compared to controls ($p = 0.22$) but not affected by the occasional or chronic frequency of use ($p = 0.85$), and the same result was obtained for FSH ($p = 0.12$ and $p = 0.85$, respectively) (see Figure S24).

Hence, no conclusive relationship could be established between LH, FSH, cannabis consumption, and steroid hormones.

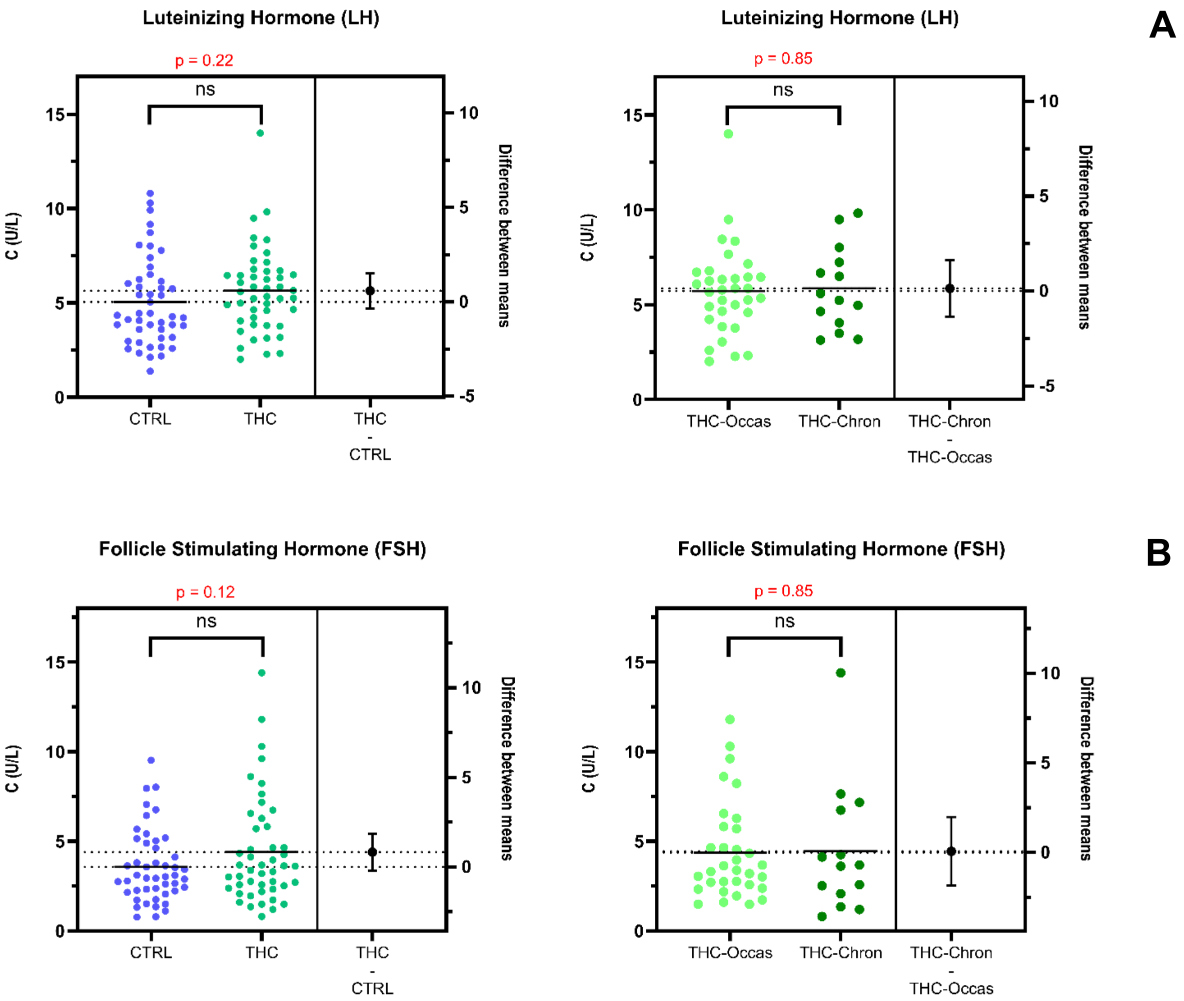

Figure S 24 : Estimation plots from univariate statistical analyses (t-tests), showing the differences in circulating levels of A) luteinizing hormone (LH) and B) follicle stimulating hormone (FSH) between controls and THC-positive participants (left pane), or occasional and chronic cannabis consumers (right pane).

Section S10: Method for THC and THC-COOH determination in serum

Serum concentrations of cannabis biomarkers (THC and THC-COOH) used for data interpretation in this study were previously measured by Zufferey et al. (*Andrology*, 2023, 10.1111/andr.13440).

The analytical procedure was as follows:

THC-D3, THC-COOH-D9, THC, and THC-COOH standards were from Cerillant (Arlesheim, Switzerland).

Reagents for the quantification of cannabinoids in blood serum were purchased from Carlo Erba (Rüti bei Büren, Switzerland) for water, acetonitrile (ACN) and methanol (MeOH) (all liquid chromatography–mass spectrometry [LC–MS] grade) or from Sigma–Aldrich (Buchs, Switzerland) for acetic acid, n-hexane, ethyl acetate and ammonium formate.

Briefly, to quantify THC and THC-COOH in serum, 250 µL of blood serum was spiked with 50 µL of a mixture of deuterated internal standards (containing 0.1 µg/mL of each THC-D3 and THC-COOH-D9). The sample was acidified with acetic acid (10% in H2O), and liquid–liquid extraction was carried out using 2.5 mL of a mixture of n-hexane/ethyl acetate (90:10, v/v) on a shaker for 10 min. After centrifugation, the organic supernatant was transferred to a separate vial and the solvents were evaporated under nitrogen at room temperature. The analytes and internal standards were reconstituted using 50 µL of a mobile phase mixture (MeOH/H2O, 1:1, v/v). Chromatographic separation was performed on an Agilent Eclipse Plus C18 column (100 × 2.1 mm, 1.8 µm) from BGB Analytik (Böckten, Switzerland), and detection was carried out on an LC–MS/MS Ultimate 3000 instrument coupled with a TSQ Quantum from Thermo. The column temperature was 50°C and the injection volume was 5 µL. The mobile phase flow rate was set at 600 µL/min. Mobile phase A was ammonium formate buffer (5 mM, pH 7.7), and mobile phase B was ACN. The elution gradient started at 30% B and reached 95% B in 5 min, after which it was held for 1 min. It was then finally decreased to 30% B and held for 3.9 min (total run time of 10 min).
