# Supplementary material for: Cannabis consumption is associated with altered steroid metabolism in young men
Source: Commun Med (Lond). 2026 Apr 16;6:224. doi: 10.1038/s43856-026-01469-x (PMC13087242; doi:10.1038/s43856-026-01469-x)
Supplement: Supplementary file 3 — Description of Additional Supplementary files [file 43856_2026_1469_MOESM3_ESM.docx]

**Description of Additional Supplementary Files**

Supplementary Data 1: List of target steroids from this work with their respective unequivocal identifiers.

Supplementary Data 2: Association of the 77 steroid compounds consistently detected in the 94 serum samples and pooled QC samples with the corresponding 13C SILs used for signal normalization.

Supplementary Data 3: Identity of the 77 steroid compounds consistently detected in the 94 serum samples and pooled QC samples, with the associated quality metrics: CV in QCs and missingness in samples.

Supplementary Data 4: Absolute concentrations (in ng/mL) of seven major steroids in serum samples from chronic and occasional cannabis users measured in this study.

Supplementary Data 5: Full LC-MS/MS dataset supporting the findings of this study, including normalized peak areas for 70 steroid metabolites passing quality checks, and phytocannabinoid levels measured in participants.

Supplementary Data 6: Numerical data plotted in Figure 1.

Supplementary Data 7: Numerical data plotted in Figure 3.
